# Does Christian Spirituality Enhance Psychological Interventions on Forgiveness, Gratitude, and the Meaning of Life? A Quasi-Experimental Intervention with the Elderly and Youth

**DOI:** 10.3390/nursrep10020022

**Published:** 2020-12-17

**Authors:** María Salvadora Ramírez Jiménez, Emilia Serra Desfilis

**Affiliations:** Faculty of Psychology, University of Valencia, 46010 Valencia, Spain; emilia.serra@uv.es

**Keywords:** religion–spirituality, Christianity, religious–spiritual prejudice, forgiveness, gratitude, meaning of life, creative visualization technique, psychoeducation, elder and youth

## Abstract

Scientific research has provided theoretical evidence on the implementation of religious/spiritual interventions (RSI) as a complementary health therapy, where spiritual improvements are also a factor to consider. Despite the above, there are few studies that have evaluated the clinical applicability of these findings. This study was an intervention with older and younger adults divided into two treatment groups and one control group. What is expected is that the two treatment groups will score better than the control group; however, the group with a Christian spiritual focus is expected to perform better than the group without a spiritual focus. Measures of gratitude, meaning of life, forgiveness, spirituality, religiosity, and expected prejudice were recorded. The hypothesis is fulfilled that Christian spirituality enhances psychological interventions on factors associated with personal well-being, mainly in older adults: spirituality (*M* = 26.00, *SE* = 2.127/*M* = 29.38, *SE* = 1.953, t (12) = −2.436, *p* < 0.05, r = 0.58), goals (*M* = 22.92, *SE* = 1.022/*M* = 24.54, *SE* = 0.739, t (12) = −2.298, *p* < 0.05, r = 0.55), and benevolence (*M* = 17.31, *SE* = 1.554/*M* = 21.08, *SE* = 1.603, t (12) = −3.310, *p* < 0.05, r = 0.69). The most powerful results of the study are those associated with religiosity/spirituality.

## 1. Introduction

### 1.1. Background

The scientific literature on health has identified the importance of studying and intervening in psychosocial processes throughout the life cycle; for example, in adolescence, the relationships between parents and children [1,2,3], somatic symptoms [4], violent and aggressive behaviors [5,6], academic performance [7], and alcohol consumption [8]. However, compared to adolescence, less attention has been paid to adult development [9]. Some important factors, such as religiosity/spirituality (R/S), could have a crucial influence on the health of older people, especially in old age and aging. Therefore, the R/S as a factor of personal well-being and protector against physical and mental illnesses must be considered in more detail in fields of knowledge such as medicine and nursing, including specific intervention programs for community application.

Although religiosity and spirituality have their own definitions, many studies agree with using R/S as a whole as a single term because one contains the other [10,11]. Koenig [12] describes how the definition of spirituality has changed and expanded over the years as researchers have examined its relationship to mental and physical health. These aspects are relevant when addressing the issue in the population, since to analyze the impact of R/S, there are substantial differences and/or difficulties when defining R/S that must be taken into account, mainly in relation to R/S itself; therefore, the modern clinical version of spirituality includes religion, positive indicators of mental health, and the secular as part of its definition. In this model, everyone is spiritual, including atheists and agnostics, so it has great clinical utility.

There are many reasons why spirituality/religiosity should be studied, since R/S beliefs and experiences are an integral part of personality [13], and because a growing line of empirical research shows important reaches of spirituality in various aspects [14]. Pargament and Mahoney [15] report that the American Psychological Association has defined religiosity as a variable of cultural diversity; therefore, the idea of the religious/spiritual dimension cannot be neglected when evaluating, diagnosing, and treating people, and future studies should include sociocultural variables (external) and evolutionary variables (internal) in their analyses, which could intervene in the process of assessment, experience, and expression of R/S, since each is a significant variable [16]. Thus, it is recognized that they are essential components of psychological baggage and not simple extensions of other needs or dispositions [10,17].

The systematic review and meta-analysis by Goncąlves et al. [18] corroborates that the scientific literature in recent decades has demonstrated the important role of R/S in people’s mental and physical health [19]. With regard to mental health, some studies have shown a direct relationship with psychological well-being [20,21]. For example, Koenig et al. [22] report a positive correlation with social support (95%), purpose and meaning in life (93.7%), and well-being, optimism, and hope (79%). In the systematic review by Gonçalves et al. [23], Larson’s findings [24] are sustained that clinical findings express the need to integrate R/S in the physical, psychological, social, and spiritual health and well-being of a person.

For Koenig [19], R/S has an impact on personal well-being and therefore, on mental health because it provides “powerful cognitions”, an optimistic world view, resources to face stress and its consequences, better coping and sense of subjective control over events, meaning and purpose of life; provides satisfactory answers to existential questions; influences the cognitive evaluation of negative life events, generating less anguish; normalizes loss and change; provides role models and opportunities for prosocial behavior, participation, and social support; enables human virtues such as honesty, forgiveness, gratitude, patience, reliability, and improvement of social relationships, both in healthy people and with some pathology or impediment. One of the characteristics of this approach is that it does not require the person to be well physically, mentally, or financially in order to carry it out. Therefore, the present study addresses the three factors of personal well-being associated with health, personal well-being, R/S, and older adults, such as gratitude [10,25,26,27,28], the meaning of life [10,29,30,31,32], and forgiveness [12,27,33].

Many studies and authors show that spirituality occupies a central position in a good aging process [34,35,36], and important benefits of R/S have been documented in health and well-being and in relieving elderly people of the stress of hospitalization and facing death, among others; furthermore, the religious and existential experience is more experiential and occurs with greater meaning when there are moments of crisis and when control over life is insecure [34,36,37,38]. This is important because it entails a series of gains that, as indicated by Koenig [19] in his review article, implies that more R/S leads to more mental health, personal well-being, and a faster adaptation to health problems compared to those that have less R/S, and that these possible benefits have physiological consequences that affect physical health and the risk of disease, and influence response to treatment. These are very important aspects to consider in the elderly population, which shows the importance of incorporating religious/spiritual themes in approaches to health in general.

Studies on R/S are generally theoretical and observational, and some health professionals have doubts about how to approach R/S in clinical practice [21]. Goncąlves et al. [18] and Gonçalves et al. [23] indicate that there are few studies that have evaluated the clinical applicability of these findings, mainly through controlled clinical trials; therefore, some authors have proposed to investigate whether the stimulation of R/S beliefs could generate better results clinical trials [39,40], since it is believed, for example, that religious/spiritual interventions (RSI) can change thoughts, promote greater acceptance of the disease, foster social support, create deeper understanding of existence, and encourage belief and faith [41,42]. That is, RSI can promote healthy behaviors and improve physical/mental health, quality of life, and personal well-being, but also encourage faith and beliefs and enhancements of the spiritual dimension. It is important to note that few studies have been carried out with a healthy population [18,23], so this study contributes to the scarce investigation of RSI in this type of population.

Gonçalves et al. [23] report that in recent decades, there has been a significant increase in the search for different types of complementary therapies with promising results when combined with intervention or conventional treatment. According to these authors, scientific research has provided important theoretical evidence of the implementation of RSI as a complementary health therapy, where the improvement of the spiritual dimension is a factor to take into account for well-being, quality of life, and the health of the person, both in people who identify themselves as belonging to a specific religious/spiritual tradition, and in those who do not, but who have the need to discuss their religious/spiritual beliefs during interventions or health treatments; a matter that generates motivation in health professionals when they find clinical and well-being improvements in their patients after addressing this issue [23]. Goncąlves et al. [18] and Goncąlves et al. [23] show that despite the different models, facilitators, and populations, among others, RSI are associated with benefits and that there is evidence of the reliability of the use of R/S as a complementary treatment.

Plante and Thoresen [38] demonstrate that spiritual tools such as prayer, attendance at religious services, appreciation of the sacredness of life, volunteering, forgiveness, gratitude, meditation, exploration of meaning, purpose and appreciation in life, and bibliotherapy, among others, can be useful resources for psychotherapists dedicated to the so-called “integrated spiritual therapy”, since the positive impact that these resources generate on the psychological state of people has been demonstrated. In this sense, the “psychology of religion and spirituality of health” occurs when the psychology of religion and spirituality approaches the processes of health–disease with a focus on promotion, prevention, and positive intervention, in accordance with the psychology of health and positive psychology [34,43].

Professionals who work with older adults, mainly mental health professionals, must be able to contemplate the spiritual resources of the people they treat or study to adequately help them with the problems that may arise during the aging process [14]. Rivera-Ledesma and Montero [36] and Rivera-Ledesma and Montero-López [44] indicate that psychologists are beginning to recognize the importance of the spirituality factor in human nature; however, including spiritual aspects in psychological treatment is a recent effort that has not yet been completely achieved, since academic psychology seems to accept spiritual aspects even more slowly than clinical psychology.

### 1.2. Objective and Hypothesis

The investigation results, the desire to provide quality care, and common sense underscore the need to integrate spirituality into patient care, as patient health and well-being are at stake. Care provider satisfaction is also at stake as they attempt to provide care that addresses the whole person: mind, body, and spirit [22]. Although it is not possible to argue about the real existence of God or what is sacred, it is possible to know about the variety of ways in which people try to discover and preserve what they consider to be sacred [15]; for this reason, it is not a question of proving the validity of the cure by faith, but rather of investigating the therapeutic or healing impact of people’s faith [45]. Therefore, the objective of this study is to analyze whether Christian spirituality enhances psychological interventions on personal well-being in older and young adults, based on the hypothesis that Christian spirituality enhances psychological intervention on personal well-being, mainly in older adults.

Regarding the intervention protocols used in previous studies, Goncąlves et al. [18] and Goncąlves et al. [23] observe two models: (a) The spiritual: this focuses on moral and spiritual values, on the belief in a “self-power”, and on facing the disease through transcendence and personal beliefs. This approach may include atheists and agnostics due to the general focus of the beliefs discussed; (b) The religious: they use the traditions of religious beliefs, such as those of Catholicism, Judaism, or Islam, to help people who already identify themselves as belonging to a specific religious tradition. The present study will contribute to the aspect of the protocols in R/S, since it manages to contemplate both models. Since it is framed in Christianity, it approaches the religious by integrating the foundations of Christian religious denominations, although it also contemplates the spiritual to be encompassed in a more general, personal, and ecumenical sense, which can be applied even to non-Christians or non-believers because the message can be assumed as a “philosophy of life”, “promoter of the transcendent”, or “enabler of contact with the Higher-Self”(however it is defined).

Another recommendation by Goncąlves et al. [18] and Goncąlves et al. [23] that was put into practice in this study was to apply measures that independently contemplate the religious and the spiritual to understand the possible relationship of the different constructions in the evaluated result. Although the intervention with a spiritual approach contemplates Christianity, the “Christian faith” is not evaluated because said spiritual approach is not directed only at people with Christian faith, since Christianity is considered to be a spiritual approach that can contribute to personal well-being regardless of the religious/spiritual position of people, as observed in nursing, which receives a great influence from Christianity, indicated by Quintero-Laverde in [46] “Nursing in the Christian world”. Furthermore, as indicated by Moberg [47], a longitudinal study found that there is a trend, regardless of gender and cohort, that people become more engaged in spiritual interests and practices as they age.

In the present study, Christian spirituality will be addressed following Smith’s approaches [48]. In addition, this spiritual approach was chosen because it is a defined approach that allows for quantitative analysis to operationalize the variables, due to the cultural and religious context of the country where the study takes place; because it is a spiritual focus that the facilitator of the intervention has experience with; and because above all, it is an approach in congruence with the aforementioned factors of personal well-being and the creative visualization technique [49,50,51].

## 2. Methods

### 2.1. Design

People of different ages were considered and an intervention methodology was applied with three comparison groups. Unlike previous studies, the control group did not receive any type of intervention [18,23]. The two treatment groups were almost identical, they only differed in the spiritual characteristic, that is, if the spiritual focus of Group A was eliminated, it would be identical to Group B. In other words, better results are not only sought compared to the control group, but also through a comparison between the two treatment groups, since if Group A obtains better results than Group B, it would be evident that it is due to the Christian spiritual approach of the first group. This would show that the Christian spiritual approach enhances the treatment in the total controlled sample by age, as in that of the young and the older adults separately, where older adults would perform best. In this way, a triangulation is achieved that allows for a refinement of the results.

### 2.2. Participants

The total non-clinical sample of the study was *n* = 105 (Group A/Intervention with Christian spirituality *n* = 37; Group B/Intervention without spirituality *n* = 36; Group C/Control group *n* = 32). The total sample of elderly people was *n* = 44 (Group A/Intervention with Christian spirituality *n* = 13; Group B/Intervention without spirituality *n* = 18; Group C/Control group *n* = 13). The older adults were mostly involved in the *Asociación Amics de la Nau Gran* (Friends of Nau Gran Association) of Valencia (Spain) and people were invited by some of these members. The total sample of young people was *n* = 61 (Group A/Intervention with Christian spirituality *n* = 24; Group B/Intervention without spirituality *n* = 18; Group C/Control group *n* = 19). The young people were first-year Psychology students from the University of Valencia. The study considered young people to make a comparison of the results obtained from them with the results of the elderly, and in this way, to observe with greater precision the impact of the intervention with Christian spirituality in the elderly.

The sociodemographic data investigated in the participants were full name, fictitious name, academic level, place of residence, contact information, past occupation (does not apply to those who currently work or young people), current occupation (does not apply to retirees or those in a similar condition), socioeconomic status, religious/spiritual beliefs, religious/spiritual practices, previous training in creative visualization techniques, ease of relaxation and visualization, considerable disability or difficulty, and physical and mental/emotional ailments. Here are the main characteristics of the participants in the study groups (see Table 1 and Table 2):

The inclusion criteria to be part of the study were that the elderly had to be over 55 years old and the young adults under 30 years old, all the participants had to have adequate mental and physical health, participants could be men or women, with or without spiritual beliefs, and participants must be able to answer the evaluations of the intervention. People with significant sensory, motor, mental, and cognitive impairments were excluded from the study. Some aspects that were taken into account so that the groups were comparable were: ease or problems with relaxing, ability to imagine, previous training in visualization, and prejudice towards religion/spirituality. Before starting the intervention sessions, the first evaluation of the prejudice on religion and spirituality was made, and the second evaluation of the prejudice on the religious and spiritual was carried out when the instruments were applied for the second time.

The study was quasi-experimental. There was manipulation of the independent variable, but the participants were not randomly assigned. However, to avoid the facilitator choosing the members of the treatment groups and also because the time availability of the participants had to be considered, the participants chose the time that best suited them (a time that was stipulated according to the availability of space), so that the members in each intervention group were generated by the participants’ choice. Regarding the circumstances that allowed for the formation of the control groups, the control group of young people was another group of first year Psychology students and the control group of older people was made up of people who were not available to participate at any time in the intervention groups, and by people who signed up later in order to collaborate with the study. In addition, it is important to mention that the intervention was given by the first author of the present study, who has the knowledge to develop what is determined for the intervention: the creative visualization technique and its sub-techniques, the Christian approach, and the other topics of personal well-being.

It is important to clarify that Goncąlves et al. [18] and Goncąlves et al. [23] indicate that some studies on RSI do not comply with the blinding of patients and facilitators (“double blind”) and that very few studies apply “third blind”, which is the blinding of the evaluator. However, this is not a problem because the modified CONSORT (Consolidated Standards of Reporting Trials) guidelines for non-pharmacological approaches do not invalidate research that does not have these aspects [52,53].

### 2.3. Place

The study complies with the ethical standards according to the Declaration of Helsinki in addition to the Consolidated Report Testing Standards (CONSORT). The requirements demanded by the *Asociación Amics de la Nau Gran* for working with its associates were completed and signed. All participants provided written informed consent, and the objective, relevance, and implication of participating in the respective intervention group were explained to them, and any doubts were clarified. The data were recruited at the Faculty of Psychology of the University of Valencia during the first session of the groups. The fictitious name provided by the participants was used in the data matrix to ensure their anonymity. The intervention process, considering all the groups, took place between February and October 2019. The elderly in Group A and Group B received the invitation through an email and a poster, with the help of the *Asociación Amics de la Nau Gran*. The young people in Group A and Group B were invited to a Psychology class at the University of Valencia.

### 2.4. Intervention

Treatment consisted of 12 sessions for both Group A and Group B (see the detailed description of the sessions in Appendix A). One session was held per week. The sessions lasted one hour and fifteen minutes each. From sessions three to eleven, the task was to practice what was developed in the session. In the next session, doubts would be clarified and a short questionnaire would be answered about the task and the previous session. The facilitator decided to give “out-of-class” assignments to apply what was developed in each session in order to promote a frequency that would allow for short-term effectiveness, as indicated by the theory on the creative visualization technique [54,55]; furthermore, in each session, brief questions were asked about the task and the previous session, in order to verify if participants practiced outside of the sessions and if they were understanding what was developed in each session. The psychoeducation of the creative visualization technique and its sub-techniques were carried out, and the aspects related to the topics of personal well-being: gratitude, meaning of life, forgiveness, and Christian spirituality (the latter for the groups that would receive this approach). The factors of personal well-being that were addressed in the intervention were addressed through the creative visualization technique.

As Gawain [54] indicates, the creative visualization technique is a state of consciousness and the art of using mental imagery and positive affirmations to explore, discover, and/or modify the deepest basic attitudes towards life. Creative visualization is not just a mental activity, it also involves the body, its emotions, and senses. It starts from the principle that the mental image precedes action and that it is the governor of behavior [49,50,55]. Creative visualization is not being absorbed in a kind of self-contemplation, but it is a process that has the ability to open or expand awareness and sensitize the needs of others beforehand [49,56]. It is used with great success in many fields, for example, health [54,55], since it exerts a physiological effect on the body. For this reason, it has begun to be used therapeutically in serious diseases such as cancer or AIDS, to everyday psychosomatic illnesses such as insomnia, headaches, or digestive problems, which is why it is part of the standard treatments in most clinics in the United States [57]. Additionally, the mind–body relationship that this technique facilitates helps towards a better adherence to treatments, a healthy lifestyle, and a favorable quality of life in general [58].

Regarding the importance of having similarity between the standard protocols and procedures [18,23], in the present study, a review of the different methods of the visualization technique was carried out, such as the Simonton Method [51,57] and the Bonny Method [59,60,61,62], to establish common criteria for an adequate application of the technique. Then, a method was chosen that was consistent with the study’s approach, that received adequate evaluations, and that had scope with the personal factors of well-being that would be developed. In addition, the topics most suggested by the authors and the recommendations of experts about the factors of personal well-being chosen were taken into account, to design the activities that allowed their development through the creative visualization technique and of the other supports used. In this way, it was possible to unify various protocols and procedures previously used, being faithful to them in order to generate evidence of their effectiveness. The Gawain [54] method met the most required criteria.

Goncąlves et al. [18] and Goncąlves et al. [23] indicate that in previous studies, RSI have been promoted through the following approaches or therapies: psychotherapy, meditation, audiovisual resources, pastoral services, and guided visualization. The present study considers criteria of three of these modalities, although the main technique is creative visualization. However, psychotherapy and audiovisual resources deepened the scope of this technique and the psychoeducational approach that the intervention has, which generates a very comprehensive protocol with a greater guarantee of effectiveness. It is important to clarify that these authors confirm that psychotherapeutics also includes the psychoeducational. As in other studies [18,23], the psychotherapeutics of the present investigation encompasses religious/spiritual values and beliefs through practical activities such as reading verses and texts from the Bible, in order to generate reflections that promote transcendence, healthier attitudes and habits, and well-being by reinforcing gratitude, forgiveness, and the meaning of life. Guided or creative visualization contemplates, like other studies [18,23], the visualization of spiritual beings that help and accompany life, also using images that refer to forgiveness, gratitude, and the purpose of life in relation to divinity and to the emotions, feelings, and activation of the senses that they generate. Regarding audiovisual resources, religious/spiritual topics are included to generate healthier attitudes and habits and well-being by inspiring gratitude, forgiveness, and a sense of life. Materials such as brochures, audio, and videos were selected and created for personal use, with the hope that they would be effective not only during the sessions but also in performing the tasks addressed in the session outside of the sessions themselves. In addition, as in the other studies [18,23], they were trained in the applied techniques and the content developed during the process was evaluated. 

### 2.5. Results

The evaluation instruments used in this research were included in a single protocol. Below is their description:

- Brief Scale of Religiosity (BSR). In Bernabé-Valero [10], some of the items used to measure the religiosity of young Spaniards by the *Santa María* Foundation in their sociological surveys and other previous psychological studies were taken as a reference, generating the BSR, which is made up of four items: Degree of religiosity, Frequency of worship attendance, Frequency of prayer, and Importance of God in one’s life. Each item has six possible answers placed ordinally, having a rating range of 4 to 24 points. In Bernabé-Valero [10], the reliability coefficients report that it obtains high internal consistency (α = 0.88, *ρ* = 0.89) and that most of the indices indicated that these four items form a latent factor with good psychometric properties. According to this author, the composite reliability obtained a value of 83% and the coefficient of variance extracted from the factor obtained a value of 55%, so it can be stated that this measure provides very good reliability indices. In the present study, Cronbach’s alpha was α = 0.89.

Religiosity is the external and social practice of a particular spiritual understanding [63,64]. This practice can be in private or in public [22]. Religiosity is an attitude made up of three dimensions: cognitive (religious beliefs), behavioral (more or less institutionalized and conventional religious behaviors and rituals), and affective (links between human beings and transcendence) [16].

- Spirituality Scale (Spiritual Acceptance vs. Rational Materialism; ST3). Extracted from the Temperament and Character Inventory—Revised (TCI-R; [65]). The Spanish version from Fernández-Aranda et al. [66] is used. It is composed of 8 Likert-type items with 5 points (1 = False, 5 = True). The rating range is from 8 to 40 points. In Bernabé-Valero [10], the goodness indices were indicated to be adequate. The reliability coefficients indicated good internal consistency (α = 0.86, *ρ* = 0.87). The composite reliability reached a value of 85%. It presented good psychometric properties, shown by the good fit indices obtained in the AFC and in the various reliability indices used. In the present study, Cronbach’s alpha was α = 0.86.

Spirituality is an intrinsic quality of the individual. It is the desire and search for a relationship with the sacred or transcendent, through any route or experience of life, and the consequent subjective/emotional experience [67]. Spirituality encompasses traditional, theocentric, and institutionally based spiritual expressions, and non-theistic expressions, apart from traditional beliefs and practice [15]. Therefore, each person has their own particular spiritual culture, which is the result of the combination of their personal spirituality and the spiritual experiences or cultural schemes to which they have been exposed [68].

- Gratitude Questionnaire—Six Items Form (GQ-6) from McCullough et al. [69]. This is composed of 6 Likert-type items with 7 points (1 = Strongly disagree, 7 = Strongly agree). The rating range is from 6 to 42 points. This is a scale for the measurement of gratitude as a dispositional trait. A confirmatory factor analysis suggested an adequate model–data fit (χ^2^(9,235) = 30.34, *p* < 0.001, CFI = 0.95, SRMR = 0.04) and the internal consistency was high (α = 0.82), so this may indicate that it has good psychometric properties, being confirmed by two other studies [10]. Chen et al. [70] eliminated item 6 from GQ-6 and confirmed a good fit of the resulting 5-item unifactorial model, obtaining a high internal consistency (α = 0.80). Bernabé-Valero [10] agrees with the previous findings and indicates that the 5-item scale is preferable because it is more parsimonious, that is, it measures the same construct satisfactorily but with a lower number of items, therefore the use of the GQ-5 scale. In the present study, the GQ-5 was used and Cronbach’s alpha was α = 0.73.

The word gratitude comes from the Latin root *gratia* which means grace, kindness, or gratitude [71]. Gratitude is a tendency to recognize, value, and respond to the positive (even negative or adverse) aspects of personal existence, experienced as gifts received [10,26]. Bernabé-Valero [10] explains that when gratitude is conceptualized as an existential attitude, there are four basic processes, which are: the recognition of gifts, the attribution of the gift to an agent, the appreciation of the gift, and the expression of gratitude. In addition, it indicates that the attitude of gratitude is shown as emotion, cognition, and behavior.

- Purpose in Life Test (PIL). Extracted from Crumbaugh and Maholick [72]. The Spanish version of García-Alandete, Martínez, and Sellés-Nohales [73], called PIL-10, is Part A of the PIL that consists of three parts. The PIL-10 is composed of 10 Likert-type items with 7 points (1 = the most “negative” option, 7 = the most “positive” option, and 4 a neutral position). The PIL-10 has a bifactorial structure: (a) Factor 1: Satisfaction and Meaning of Life (SML), composed of 6 variables (1, 2, 4, 7, 8, and 9) and related to the general perception of meaning of the life; (b) Factor 2: Goals and Purposes in Life (GPL), composed of 4 variables (3, 5, 6, and 10) and related to specific life goals. The rating range for SML is 6 to 42 points, and for GPL, it is 4 to 28 points. A higher total score indicates a higher achievement of meaning. In García-Alandete et al. [74], an adequate data–model fit was obtained, supporting its structure (χ^2^S-B = 101.0105, df = 34, *p* < 0.01; CFI = 0.929, RMSEA = 0.066), with a high correlation between the factors (r = 0.734, *p* < 0.01). The adequacy of this structure has been confirmed in the work by Martínez et al. [75] (NFI = 0.923, GFI = 0.946, AGFI = 0.912, CFI = 0.934, SRMR = 0.041, RMSEA = 0.081). In Bernabé-Valero [10], the goodness indices indicated a good fit of the model. The reliability coefficients reported good internal consistency (*α* = 0.85, ρ = 0.86). The composite reliability obtained a value of 76% for factor 1, being considered acceptable, and 67% for factor 2, with a value very close to the recommended one. The correlation between the factors was significant, reaching a value of 0.86. Therefore, adequate psychometric properties of this scale were guaranteed. In García-Alandete et al. [76], the scale showed high internal consistency (α = 0.86), as well as the SML factor (α = 0.83), although the consistency of the GPL factor was acceptable (α = 0.71). In the present study, Cronbach’s alpha for SML was α = 0.86 and Cronbach’s alpha for GPL was α = 0.82.

The meaning of life for Frankl [77] is to find a purpose (goals and objectives) that gives motivation, courage, satisfaction, encouragement, and love, generating the conviction in the person that life always has meaning, even in difficult and unpleasant moments, as for example in suffering, because “He who has a why to live for can bear almost any how” [78], p. 35. The search for meaning is an intrapersonal process that entails freedom and responsibility, since it considers “living with decision” and the “resilient attitude” to find a higher, deeper, and hopeful meaning of life as central components [79]. As Frankl [77] indicates, the drive to search for meaning is the primary and most powerful force for motivating and guiding people’s behavior.

- The Transgression Related Interpersonal Motivations (TRIM-18). From McCullough et al. [80]. This is a Likert-type scale with 18 items (1 = strongly disagree and 5 = strongly agree). The TRIM-18 measures reasons for not forgiving (motivation for revenge and avoidance) and reasons for forgiveness (motivation for benevolence). It distinguishes 3 subscales [81,82], which are: (a) Avoidance, which uses 7 items (2, 5, 7, 10, 11, 15, and 18) to measure motivation to avoid the aggressor; (b) Revenge, which uses 5 items (1, 4, 9, 13, and 17) to measure the motivation to seek revenge; (c) Benevolence, which uses 6 items (3, 6, 8, 12, 14, and 16) to measure the motivation for benevolence. The rating range for Avoidance is 7 to 35 points, for Revenge it is 5 to 25 points, and for Benevolence it is 6 to 30 points. In the study by Serrano-Fernández [11], this scale showed adequate internal consistency, with indices of 0.92 for the Avoidance subscale, 0.88 for the Revenge subscale, and 0.87 for the Benevolence subscale. Fernández-Capo et al. [83] indicated that the scale has adequate psychometric properties for use in the Spanish population. Confirmatory factor analysis revealed a three-dimensional structure and Cronbach’s alpha values varied between 0.80 and 0.90, and those obtained for test–retest reliability varied between 0.74 and 0.84. In the present study, only the avoidance and benevolence subscales are used due to the emphasis of the forgiveness sessions in the intervention; therefore, for the present study, Cronbach’s alpha for Avoidance was α = 0.86 and Cronbach’s alpha for Benevolence was α = 0.85.

When a transgression is perceived and the responses (cognitions, memories, emotions, and behaviors) towards the offender, the offense, or its consequences are transformed from negative to neutral or positive (feelings of love or compassion are not necessary, it would be enough to abandon the negative emotions), this is defined as forgiveness [84]. It is a freely chosen resource to manage or overcome the discomfort caused by the offense [85].

- Expected and Unexpected Religious/Spiritual Prejudice (EURSP) was developed *ad hoc* to assess the prejudice that participants had about religion and spirituality. Participants were asked to associate religion and spirituality with a series of indicated terms. Study participants may not associate religion or spirituality with each term related to prejudice, so there are multiple possible answers. The questionnaire considers two factors: the expected religious/spiritual prejudice and the unexpected religious/spiritual prejudice, the first one obtained a Cronbach’s alpha of α = 0.79 and considers the terms institutional, external/objective, old, structured, static/frozen, and bad towards religion and the terms individual/personal, internal/subjective, new, functional, flexible/dynamic, and good towards spirituality [86]. The qualification range for the expected religious/spiritual prejudice factor (understand in the results “Expected prejudice”) is from 0 to 12 points.

Reductionist contrasts between religiosity and spirituality should be avoided, for example, conceiving religiosity as institutional, external/objective, old, structured, static/frozen, and bad, and spirituality as individual, internal/subjective, new, functional, flexible, and dynamic [86]. These contrasts are prejudices towards the R/S because they are based on a previous or premature judgment (*praejudicium*), which according to Cañero and Solanes [87], can be both favorable and unfavorable, although it is usually defined as a negative attitude and as a social norm characteristic of a particular culture or society.

### 2.6. Sample Size

Following the principle of intention to treat, the decision was made to include all those people who completed the process even if they had missed the sessions many times and to include people with different religious/spiritual creeds or without any religious/spiritual creed in the list of participants. All the above considerations contribute to generating greater assurance that the intervention is effective. With respect to the elderly, two participants from Group B and one participant from Group C were excluded from the analysis because they did not complete all the required instruments; with regard to the young participants, one participant from Group A, one participant from Group B, and 16 participants from Group C were excluded for the same reason.

### 2.7. Similarity of the Intervention

The information in Appendix A shows that Groups A and B (elder and youth) are similar in the number of sessions, session structure, techniques, and topics about factors of personal well-being (forgiveness, gratitude, and meaning of life). The only difference between the two treatments is that in Group A it is approached with a Christian spiritual approach, while in Group B it is not approached with a spiritual/religious approach.

The psychoeducational and personal well-being sessions had a defined structure. In the psychoeducational sessions, the structure at the beginning included a welcome and a brief approach to the task, then psychoeducation about the technique and the performance of the technique by the facilitator, and then, the individual execution of the technique by the participants. The session ended with clarification of doubts, additional comments, and the closing of the session. In contrast, in the sessions about personal well-being, the structure first included a welcome and a brief approach to the task; then, a brief psychoeducation on the topic established for the session; a practical part, which consisted of applying the breathing techniques/visual relaxation, creative visualizations, and positive affirmations on the topic to be addressed; and finally, the experience was shared and the session was dismissed.

For Group A of young and older adults, in sessions three to eleven, verses/biblical texts related to the topic developed in the session were delivered, and the image of God in this regard was addressed (this was developed in the psychoeducational part of the session and in the execution of the creative visualization technique). In addition, pieces of music of Gregorian [88] chants were heard in the background in all sessions. Gregorian music was selected because for the context, it is representative of Christianity. From the third to the eleventh session, a brochure was provided on the topic developed (in the sessions on personal well-being factors, two brochures were delivered—one on the theoretical aspects and the other on the practical aspects of the subject in question). In the first session, participants were told that the workshop topics would be addressed with a Christian spiritual approach, so participants knew in advance that if they did not agree or did not wish to participate in the study, they could discontinue participation.

In all the sessions for Group B of young and older adults, well-known pieces of instrumental music—Spanish guitar—were played in the background [89]. Spanish guitar music was selected because it is music that is considered familiar. Like Group A, from the third to the eleventh session, a brochure was provided on the topic developed (in the sessions on the factors of personal well-being, two brochures were delivered, one on the theoretical aspects and the other on the practical aspects of the subject matter; obviously in this group, the information concerning Christian spirituality was omitted).

One email was created for Group A and another for Group B for young adults, and for the older adults from Group A and Group B, a single email was created, in order to maintain order on the information that could be exchanged and to be able to complete missing information in the questionnaires/instruments.

Group C, or the control group, had participants similar to Group A and Group B and the same data were taken from the previous groups, both in the elderly and in the young adults. They did not receive intervention; only the same instruments from Group A and Group B were administered to them at two different times. The second administration occurred approximately three months after the first administration. In the elderly group, the participants were obtained through the first list of people interested in the study who could not be part of the treatment groups and through the elderly participants in the treatment groups. The group of young people was a first-year group of Psychology students from the University of Valencia, different from the course of students that made up Group A and Group B of the study.

With respect to the control group of older adults, two psychoeducational sessions on the study topics (excluding the spirituality topic) were offered as a token of gratitude, weeks after the participants filled in the questionnaires/instruments for the second time. Young people in the two treatment groups (A and B) would receive a point and a half in a grade for a course if they complied with everything indicated in the intervention.

### 2.8. Statistical Methods

Statistical analysis was performed with the IBM SPSS Statistics v25 package for Windows (New York, NY, USA) [90]. Statistically significant results are considered when *p* ≤ 0.05. A semantic content analysis and a second order factor were performed to assess the quality of the EURSP.

The comparison of the three study groups—Group A/Intervention with Christian spirituality, Group B/Intervention without spirituality and Group C/Control Group—was carried out through the Multivariate Analysis of Covariance (MANCOVA). The dependent variables were: expected religious/spiritual prejudice (understood in results as expected prejudice), religiosity, spirituality, gratitude, satisfaction and sense of life (understood in results satisfaction), goals and vital purposes (understood in results goals), avoidance, and benevolence. Age (young and older) was used as a covariate. This analysis mainly allows us to know if Group A scores better than Group B and Group C.

The comparison between before and after the intervention for the groups of older people (Group A, B, and C) and for the groups of young people (Group A, B, and C) was carried out through the F test of repeated measures. This analysis mainly allows us to know if the A groups (young and older separately) score significantly better after the intervention, in contrast to the B and C groups. In addition, it allows to know the results of groups A, B, and C according to age (young and older).

## 3. Results

### 3.1. Evaluation of the EURSP Questionnaire

A principal component analysis was performed with orthogonal rotation, since the correlations between items ranged from 0.0 to 0.6. In the final solution, the eigenvalues greater than 1 showed the existence of two factors. This solution converged in one iteration and explained 76.74% of the variance. The items present factorial loads greater than 0.30 within their factor and communalities greater than 0.35. The instrument was confirmed by 24 reagents. Bartlett’s sphericity test was significant (90.642, df = 6, Sig. < 0.001) and the Kaiser–Meyer–Olkin sample size adequacy indicator of sampling adequacy was 0.46.

### 3.2. Evaluation of the Intervention by Treatment

The MANCOVA was performed to determine the effect of the intervention on gratitude, satisfaction, goals, avoidance, benevolence, expected prejudice, religiosity, and spirituality while controlling for age. The main effects of the Wilks intervention, Λ = 0.636, F (20,184) = 2.340, *p* < 0.05, multivariate η^2^ = 0.203, indicate a significant effect on the combined dependent variables. The age covariate significantly influenced the combined dependent variables Wilks’ Λ = 0.650, F (10,92) = 4.961, *p* < 0.05, multivariate η^2^ = 0.350.

The following are the statistical results of the MANCOVA (see Table 3):

The results indicate that the intervention significantly affected the variables of expected prejudice, religiosity, and spirituality; the age covariate affected the variables of expected prejudice, religiosity, spirituality, satisfaction, goals, and benevolence.

Here are the statistical results of the multiple comparisons (see Table 4):

The above comparisons mean that the participants in the intervention with Christian spirituality have less expected prejudice than the participants in the intervention without spirituality and the participants in the control group; and that participants in the intervention without spirituality have less expected prejudice than participants in the control group. Furthermore, these comparisons show that the participants in the intervention with Christian spirituality were more religious than the participants in the control group. Finally, these results mean that the participants in the intervention with Christian spirituality and those in the intervention without spirituality were more spiritual than those in the control group.

### 3.3. Evaluation of the Intervention by Treatment and Age

Below are the results of each intervention group in older and younger adults separately (see Table 5 and Table 6):

The older adults in the intervention with Christian spirituality experienced less spirituality, goals, and benevolence before the intervention than after it, being the only group with statistically significant results between before and after the intervention.

The above results indicate that the youth in the intervention with Christian spirituality experienced less gratitude and satisfaction before the intervention than after the intervention. Young people in the intervention without spirituality experienced less gratitude and satisfaction before the intervention than after the intervention; and they experienced more avoidance before the intervention than after it. Furthermore, the youth in the control group experienced less satisfaction and goals before the intervention than after the intervention.

## 4. Discussion

### 4.1. Limitations

Due to the space availability in the Psychology Faculty, the day and time of the interventions was stipulated, with people having to adjust to these schedules and selecting which of the two possible schedules they could attend according to their schedules. Instruments that fit the study were chosen; however, it was also necessary to bear in mind that there were not many and/or they were not very long, based on the recommendation by some experts that most older people do not like to fill out questionnaires, which was verified in the present study.

### 4.2. Interpretation

It is important to highlight that discussing RSI studies is very complicated, since as shown by the systematic reviews and meta-analyses by Goncąlves et al. [18] and Goncąlves et al. [23], the studies are very diverse in methodologies, protocols, techniques, topics, results, among others. Furthermore, Lucchetti et al. [91] report that there is a lack of consensus in the studies on R/S and that this makes it difficult to compare the results between them. However, Goncąlves et al. [18] and Goncąlves et al. [23] show that despite the different models, facilitators, and populations, among others, RSI are associated with benefits (comparing the results between the pre and post intervention groups and the control groups) and that there is evidence of reliability of the use of R/S as a complementary treatment; that is, although the religious/spiritual has its negative effects, it is generally associated with greater well-being, and better mental and physical health [45], as has also been reflected in the results of the present study. Therefore, following the recommendations of Gonçalves et al. [23] so that comparison is possible, the results of the present study will be contrasted with previous studies that are as similar as possible in terms of topics and techniques.

The present study has analyzed whether Christian spirituality enhances psychological intervention on personal well-being in older and young adults, fulfilling the hypothesis that Christian spirituality enhances psychological intervention on personal well-being mainly in older adults. The most important result of the study is that Christian spirituality affected the personal well-being factors associated with the spiritual/religious dimension, since the group that received the intervention with Christian spirituality had less religious/spiritual prejudices, compared to the group with intervention without spirituality and the control group. Additionally, the Christian spirituality group was the study group that was more religious compared to the control group. Furthermore, together with the group with the intervention without spirituality, they were more spiritual than the control group, and although there were no statistically significant differences between the two treatment groups in religiosity and spirituality, the group that received the intervention with Christian spirituality he had differences of averages that favor him in both factors of personal well-being. 

The older adults who received the intervention with Christian spirituality obtained statistically significant results in spirituality, goals (factor related to meaning of life), and benevolence (factor related to forgiveness). These results reflect the approach that was carried out in each session, that is, they reflect the emphasis that each topic obtained. For example, in the sessions on meaning of life, importance was given to generating ideal goals and situations, and so the factor “goals” was significant. Thus, the intervention with a Christian spiritual approach impacts spirituality, the meaning of life, and forgiveness in older adults, making it viable as a treatment to improve the personal well-being of older adults. The impact of the approach in healthy older adults -becoming a preventive intervention and strengthening of well-being and health in said age group-, is a very relevant finding because it reinforces the evidence that spirituality occupies a central place in a good aging process [34,35,36], putting in “check” the argument that the religious and existential experience is more experiential and with greater meaning when there are moments of crisis and when control over life is insecure [34,36,37,38].

The group that received the intervention with Christian spirituality was the only one that obtained statistically significant differences between before and after the intervention in some of the factors of personal well-being, while in the group that received the intervention without spirituality and in the control group, there were no statistically significant differences between the before and after intervention in any of the variables.

As corroborated in the present study, it is important to address religious and spiritual prejudice so that the impact of the intervention is effective and positive [29,86]. The participants who received the intervention with Christian spirituality were the only ones who received psychoeducational therapy on the Christian religious/spiritual topics, generating a decrease in prejudice and an increase in religious/spiritual feelings, by assessing the positive consequences that these media generate, while the participants of the intervention without spirituality, having not received this approach, did not have statistically significant results in prejudice and religiosity because there was nothing that directly affected these aspects. Although the participants who received the intervention without spirituality obtained statistically significant results in spirituality compared to the control group, this is due to the fact that in this group the spiritual issue arose by the participants, so it had to be subtly addressed without reference to any spiritual or religious doctrine. This is why the measure of spirituality contains by itself the increase in this factor of well-being after the intervention. While the participants in the Christian spirituality intervention did receive a spiritual approach focused on Christian topics, this factor was distributed in the religious and spiritual constructs, since for many people, they are not different constructs or Christian topics are considered to be both spiritual and religious [10,11], especially since the instrument that measures religiosity directly emphasizes elements or constructs associated with a defined religion such as Christianity [10]; therefore, this also explains why the group without spiritual intervention, despite not having statistically significant differences in regard to religiosity with the group that did obtain the Christian spiritual approach, did not obtain statistically significant differences with the control group either, while the group with the Christian spiritual intervention obtained statistically significant differences with the control group with respect to religion and obtained a higher mean in religiosity than the intervention group without spirituality. Therefore, the Christian religious/spiritual approach is evident in the group that received it; furthermore, the previous data are corroborated by the fact that the control group obtained less favorable results in prejudice, spirituality, and religiosity compared to the two intervention groups, mainly with the intervention group with Christian spirituality. This is why we agree with Goncąlves et al. [18] and Goncąlves et al. [23] that it is vital to apply measures that independently contemplate the religious and the spiritual to understand the possible relationship between the different constructs in the evaluated result, because although theoretically, they are considered as a single term, it is important to evaluate the weight of each construct according to the evaluation of the participants, as recorded by the BSR and ST3 measures.

These results are important for scientific impact and for clinical application, since these results corroborate those indicated by Captari et al. [92] in their meta-analysis, which show that psychotherapy adapted to R/S resulted in a greater improvement in psychological functioning compared to control groups or with treatments without R/S; furthermore, the data obtained in more rigorous studies indicated by said authors are corroborated, where psychotherapies adapted to R/S were equally effective as standard approaches to reduce psychological distress but resulted in greater spiritual well-being, which is reflected in the results of the group with a Christian spiritual approach, both in the total sample and in the group of older adults.

The present study also shows that the statistically significant results specifically on religiosity are positive, since according to Krause [93] and Moberg [47], religious beliefs and activities, that is, practicing a religion, generates a personal adjustment in old age that leads to a greater sense of life and therefore, higher levels of self-esteem, life satisfaction, and optimism. In addition, the result obtained in the present study on religiosity is relevant because, as indicated in a study on religiosity and health in Europe carried out by Ahrenfeldt et al. [94], praying, participating in a religious organization, and receiving religious education are associated with good health compared to just praying.

It is necessary to clarify that although no religious institution was represented in the group with a Christian spiritual approach, it could be assumed by the participants as a group of religious/spiritual nature by clearly having a Christian spiritual approach. Therefore, the benefits generated by religion and religious participation can also be reflected in the process of this intervention. This is relevant because, as Steinhorn et al. [95] found, a significant number of patients do not have a strong religious community to turn to or do not belong to an organized faith community, so fostering the exploration of this dimension in the clinical and health field can help to provide context; because this space achieves similar effects to those generated by traditional religious nuances, since it is possible to address health problems, improve well-being and the feeling of well-being,. It is important to highlight and publicize these effects, especially since patients may hide it for fear of receiving disapproval from their doctors. Therefore, it is important to highlight that the data in the present study on this topic are relevant because there is evidence that shows that having higher levels of spirituality and religiosity generate better results than just having one or none of them; additionally, higher levels of religiosity compared to higher levels of spirituality were also associated with better results [96,97].

The intervention with Christian spirituality is more effective in the elderly population than in the young population, since the former had more favorable results than the latter, even in comparison with the intervention without spirituality and with the control group of elderly people. This is due to the fact that older people tend to lean more toward an “intrinsic religious orientation”, while younger-aged people tend more to a “religious search orientation” [29], and immerse themselves in the stage of questioning their primary conceptions, so they could be very critical and/or resistant to conventionalisms or traditional conceptions, although age and the development of faith do not always go hand in hand (Fowler, 1991, cited by Torres-Jiménez [32]).

The results of the present study corroborate what was evidenced by Moberg [47] that older adults obtain higher scores on measures of religion and spirituality, possibly because they delve deeper into spiritual interests and concerns and because more religious and spiritual people tend to live longer than others. In addition, older adults are more receptive and interested in the spiritual aspects that the themes evoke, when compared to young people [14,98]. Moberg [47] mentions that in a longitudinal study, a trend was found, regardless of gender and cohort, that as people age, they become more engaged in spiritual interests and practices, which would explain the emergence of the spiritual theme in the treatment group of older adults without a spiritual focus.

These data are also very relevant because they coincide with the findings of the longitudinal study by Kent et al. [98], where forgiveness, attachment to God, and mental health are addressed; these authors indicate that older people are more reflective about their relationship with God and use their remaining years to expel guilt and resolve intrapsychic conflicts, that is, the type of attachment they have with God and the possibility of forgiving generates an increase in mental health compared to younger people, and that this is related to the desire to give meaning to their lives and to form experiences and relationships in a coherent whole; furthermore, attending religious services enables greater optimism over time.

Vaillant [99] considers that in old age, there is not necessarily an increase in spirituality, and that this does not favor the way of aging. This author suggests that “aging gracefully” is related to six tasks: (1) Caring for others, being open to new ideas, and staying socially useful; (2) Maintaining integrity posited by Erikson, that is, accepting the past and spending moments in the past to sustain achievements and being open to learn from the next generation; (3) Maintaining the other strengths of Erikson’s theory, such as hope in life, striving to be as autonomous as possible, and the value of all initiative; (4) Enjoying life, encouraging a sense of humor and a willingness to play; (5) Being tolerant with the unpleasant aspects of old age, taking care of yourself, accepting dependency needs, and appreciating the support received; and (6) Maintaining contact and intimacy with old friends and making new ones. However, the results of the present study agree with Tornstam [100] and San Martín-Petersen [14] that many of the concepts that the author associates with “aging gracefully”, for example, kindness, generosity, tolerance, gratitude, and hope, are aspects that other authors consider as factors or characteristic elements of spirituality. The important factor is that aging has its own meaning and nature, and that there is a continuous development in old age characterized by change and not by stability and continuity, so to discover these neglected or hidden aspects of old age, it is essential to listen to what older adults have to say.

In the specific results of the groups of young people, there was an impact of Christian spirituality on factors of personal well-being, since the group that received the intervention with Christian spirituality obtained statistically significant differences between before and after the intervention in gratitude and satisfaction, obtaining an increase in these two factors; similarly, the group that received the intervention without spirituality also obtained statistically significant differences in gratitude, satisfaction, and avoidance, obtaining an increase in the first two and a decrease in the last factor mentioned; however, the control group also showed statistically significant results between before and after the process in satisfaction and goals, obtaining better results in the second application of the instruments. Therefore, the result of the satisfaction factor may be due more to the feeling generated by the completion of a school year, since the second application of the instruments coincided with the end of the semester, while the gratitude and avoidance factors would be associated with the intervention since it occurs in the two treatment groups and not in the control group, showing that both approaches are equally effective in addressing the gratitude factor in the young population, while the intervention without spirituality seems more effective in addressing avoidance in said age population.

The intervention without spirituality was slightly more effective for young people compared to their counterparts from the other intervention group, since it seems that they felt more comfortable in the group and possibly religious/spiritual prejudices—which are considerable and greater in the young population than in the older population—could be activated less in this group than in the group that received the Christian spiritual focus, since they did not have to interact with these issues. This is relevant considering that the young people are mostly non-believers; however, an intervention with Christian spirituality also generates important results in young people and its impact does not differ considerably from interventions without a Christian spiritual approach. In older adults, the spiritual approach, in this case, Christian spirituality, further enhances the intervention, despite the fact that it may not be sufficiently valued by this age group. In summary, these results show that the intervention with Christian spirituality impacted the elderly more than the intervention without spirituality, and that in the young participants, it did have an impact, so important evidence of this study is that the positive effect of Christian spirituality is not affected by the diversity of religious/spiritual positioning, nor is it associated with a single stage of the life cycle, that is, it is beneficial both in people who identify themselves as belonging to a specific religious/spiritual tradition, and in those who do not belong to a specific tradition but have the need to discuss their religious/spiritual beliefs during health interventions or treatments [23].

It is important to bear in mind that, following the modern clinical version of spirituality by Koenig [12], the study groups were made up of a theistic and atheistic population, with and without spiritual beliefs, with religious positions and without religiosity. These characteristics of religious/spiritual positioning highlight the statistically significant results, since it reinforces that the intervention with a Christian spiritual approach has been effective, since despite this diversity, it had an impact. One aspect of the spiritual focus that should be explained is that it was not applied as a “recipe”, since the structure of the sessions and the technique used allowed for a group approach with a private and personal focus, as recommended by Gonçalves et al. [23], who indicate that the choice of RSI should be made in agreement between the health professional and those who receive the care.

The role of the technique in the development of the topics is highlighted, since it is associated with the best results obtained by groups A and B compared to the control groups [18,23]. In agreement with Araújo-Elias et al. [101], this technique does not provide a cognitive or rational impact, but rather, the ability to recognize one’s own potential and energy force, and the possibility of being able to build a better and more integrated life; this would corroborate what it indicates Gawain [54], which is that Creative Visualization, more than a technique, is a state of consciousness that favors the improvement of well-being and could channel spiritual awakening in the medium/long term. This would also explain the statistically significant results obtained in spirituality by the two treatment groups, since the technique also contributed to generating this result in both groups.

On the other hand, unlike the results mentioned in the systematic reviews and meta-analyses of Goncąlves et al. [18] and Goncąlves et al. [23], a very important finding of the present study is that the effect sizes were not small, since the statistically significant results corresponding to the F Test of repeated measures show higher results than r = 0.30 (mean) and r = 0.50 (large), which indicates that in some cases, the effect implies 9% of the total variance and in other results, the effect implies 25% of the total variance.

Finally, it is important to highlight the efforts made in this research to try to improve the methodological problems of other R/S works, which has contributed to generate, as indicated by Goncąlves et al. [18], the most credible and reliable answers to questions on this topic.

## 5. Conclusions

Christian spirituality enhances psychological intervention on personal well-being, mainly in older adults. In addition, the intervention had its impact despite the diversity of beliefs, prejudices, resistance, and assistance, so that the effectiveness of the Christian spiritual approach in psychological intervention on personal well-being is not nullified nor is its approach inadequate, being reliable as a complementary treatment. Therefore, how to properly incorporate clinical implications and professional conduct in the religious/spiritual field into the study program should be analyzed.

## Figures and Tables

**Table 1:** Sociodemographic data.

Groups	Participants	Age	SD	Female	Male
Older adults					
Group A	13	69.26	5.13	12	1
Group B	18	68.09	4.10	14	4
Group C	13	68.83	5.36	11	2
Young adults					
Group A	24	19.21	1.10	23	1
Group B	18	19.45	1.26	17	1
Group C	19	18.98	0.80	14	5

Except for age, the other data are reported in frequencies.

**Table 2:** Religious/spiritual characteristics of the participants.

Groups	Profess Christianity	They Do Not Profess Christianity	They Are Interested in the Sacred/Spiritual	They Are More or Less Interested in the Sacred/Spiritual	They Are Not Interested in the Sacred/Spiritual
Older adults					
Group A *n* = 13	12	1	8	5	0
Group B *n* = 18	11	7	7	6	5
Group C *n* = 13	4	9	4	4	5
Young adults					
Group A *n* = 24	10	14	6	7	11
Group B *n* = 18	5	13	6	7	5
Group C *n* = 19	6	13	3	8	8

Data are reported in frequencies.

**Table 3:** Statistical results of the MANCOVA (Intervention with Christian spirituality *n* = 37; Intervention without spirituality *n* = 36; Control group *n* = 32).

Independent Variable and Covariate	Dependent Variables	F (df between, df Error)	*p*-Value	η^2^ Partial
Intervention				
	Expected prejudice	11.956 (2,101)	<0.05 *	0.191
	Religiosity	6.974 (2,101)	<0.05 *	0.121
	Spirituality	6.500 (2,101)	<0.05 *	0.114
	Gratitude	2.137 (2,101)	>0.05	0.041
	Satisfaction	0.950 (2,101)	>0.05	0.018
	Goals	0.011 (2,101)	>0.05	0.000
	Avoidance	0.870 (2,101)	>0.05	0.017
	Benevolence	1.899 (2,101)	>0.05	0.036
Age covariate				
	Expected prejudice	9.063 (1,101)	<0.05 *	0.082
	Religiosity	15.811 (1,101)	<0.05 *	0.135
	Spirituality	6.638 (1,101)	<0.05 *	0.062
	Gratitude	1.218 (1,101)	>0.05	0.012
	Satisfaction	16.193 (1,101)	<0.05 *	0.138
	Goals	4.779 (1,101)	<0.05 *	0.045
	Avoidance	3.070 (1,101)	>0.05	0.030
	Benevolence	5.699 (1,101)	<0.05 *	0.053

* Statistically significant results are considered when *p* ≤ 0.05.

**Table 4:** Statistical results of multiple comparisons (Intervention with Christian spirituality *n* = 37; Intervention without spirituality *n* = 36; Control group *n* = 32).

Groups	Statistics	EP	R	Sy	Ge	Sn	Gs	A	B
Group A and Group B	t (df error)	−2.65 (101)	1.86 (101)	1.22 101)	−0.34 (101)	0.45 (101)	−0.15 (101)	−0.54 (101)	0.69 (101)
	*p*-value	<0.05 *	>0.05	>0.05	>0.05	>0.05	>0.05	>0.05	>0.05
Group A and Group C	t (df error)	−4.89 (101)	3.72 (101)	3.48 (101)	1.47 (101)	1.32 (101)	−0.1 (101)	−1.30 (101)	1.89 (101)
	*p*-value	<0.05 *	<0.05 *	<0.05 *	>0.05	>0.05	>0.05	>0.05	>0.05
Group B and Group C	t (df error)	−2.84 (101)	2.32 (101)	2.70 (101)	2.02 (101)	1.04 (101)	0.04 (101)	−0.92 (101)	1.44 (101)
	*p*-value	<0.05 *	>0.05	<0.05 *	>0.05	>0.05	>0.05	>0.05	>0.05

Group A—Intervention with Christian spirituality; Group B—Intervention without spirituality; Group C—Control group. EP—Expected prejudice; R—Religiosity; Sy—Spirituality; Ge—Gratitude; Sn—Satisfaction; Gs—Goals; A—Avoidance; B—Benevolence. * Statistically significant results are considered when *p* ≤ 0.05.

**Table 5:** Statistical results of the repeated measures F test in older adults (Intervention with Christian spirituality *n* = 13; Intervention without spirituality *n* = 18; Control group *n* = 13).

Dependent Variables and Groups		Before the Intervention		After the Intervention				
		Mean	SE	Mean	SE	t (df between)	*p*-Value	r
EP	Group A	5.69	1.028	5.77	0.833	−0.067 (12)	>0.05	0.02
	Group B	7.56	0.612	6.67	0.626	1.315 (17)	>0.05	0.30
	Group C	6.54	1.048	8.23	0.769	−1.923 (12)	>0.05	0.49
R	Group A	14.23	1.226	14.31	1.190	−0.158 (12)	>0.05	0.05
	Group B	11.61	1.353	12.00	1.255	−0.693 (17)	>0.05	0.17
	Group C	8.62	1.546	8.31	1.327	0.652 (12)	>0.05	0.18
Sy	Group A	26.00	2.127	29.38	1.953	−2.436 (12)	<0.05 *	0.58
	Group B	26.28	1.977	25.11	2.174	1.356 (17)	>0.05	0.31
	Group C	21.31	2.205	19.69	2.588	1.363 (12)	>0.05	0.37
Ge	Group A	29.31	1.100	30.77	0.914	−1.206 (12)	>0.05	0.33
	Group B	31.06	0.818	32.11	0.661	−1.513 (17)	>0.05	0.34
	Group C	30.31	1.298	30.46	1.233	−0.205 (12)	>0.05	0.06
Sn	Group A	34.15	1.409	34.69	1.046	−0.520 (12)	>0.05	0.15
	Group B	35.22	0.858	34.67	1.032	1.033 (17)	>0.05	0.24
	Group C	36.85	1.427	35.38	1.101	1.731 (12)	>0.05	0.45
Gs	Group A	22.92	1.022	24.54	0.739	−2.298 (12)	<0.05 *	0.55
	Group B	24.72	0.713	24.06	0.618	1.258 (17)	>0.05	0.29
	Group C	25.23	0.717	24.46	0.637	1.146 (12)	>0.05	0.31
A	Group A	21.92	1.323	21.38	1.670	0.348 (12)	>0.05	0.10
	Group B	24.50	1.826	22.61	2.065	1.420 (17)	>0.05	0.33
	Group C	23.85	1.931	24.08	2.108	−0.090 (12)	>0.05	0.03
B	Group A	17.31	1.554	21.08	1.603	−3.310 (12)	<0.05 *	0.69
	Group B	22.44	1.263	20.89	1.536	1.276 (17)	>0.05	0.07
	Group C	17.77	1.537	17.15	2.215	0.237 (12)	>0.05	0.07

Group A—Intervention with Christian spirituality; Group B—Intervention without spirituality; Group C—Control group. EP—Expected prejudice; R—Religiosity; Sy—Spirituality; Ge—Gratitude; Sn—Satisfaction; Gs—Goals; A—Avoidance; B—Benevolence. * Statistically significant results are considered when *p* ≤ 0.05.

**Table 6:** Statistical results of the repeated measures F test in young adults (Intervention with Christian spirituality *n* = 24; Intervention without spirituality *n* = 18; Control group *n* = 19).

Dependent Variables and Groups		Before the Intervention		After the Intervention				
		Mean	SE	Mean	SE	t (df between)	*p*-Value	r
EP	Group A	7.13	0.549	7.08	0.611	0.080 (23)	>0.05	0.16
	Group B	8.39	0.472	8.61	0.567	−0.524 (17)	>0.05	0.13
	Group C	8.53	0.609	9.74	0.512	−1.898 (18)	>0.05	0.41
R	Group A	8.21	1.053	8.04	1.099	0.526 (23)	>0.05	0.11
	Group B	8.06	0.965	7.61	1.004	1.365 (17)	>0.05	0.31
	Group C	7.53	0.912	7.68	0.955	−0.528 (18)	>0.05	0.12
Sy	Group A	19.54	1.655	21.17	1.681	−1.949 (23)	>0.05	0.38
	Group B	20.67	1.736	22.00	1.730	−1.609 (17)	>0.05	0.36
	Group C	18.84	1.421	18.79	1.515	0.075 (18)	>0.05	0.02
Ge	Group A	29.04	0.781	30.79	0.684	−2.708 (23)	<0.05 *	0.49
	Group B	29.28	0.939	30.78	0.726	−2.352 (17)	<0.05 *	0.50
	Group C	27.95	1.294	29.53	0.880	−1.448 (18)	>0.05	0.32
Sn	Group A	28.54	1.274	30.17	1.220	−2.291(23)	<0.05 *	0.43
	Group B	27.06	1.454	31.56	1.384	−3.830 (17)	<0.05 *	0.68
	Group C	28.16	1.503	31.00	1.049	−2.378 (18)	<0.05 *	0.49
Gs	Group A	21.58	0.814	22.17	0.711	−1.143 (23)	>0.05	0.23
	Group B	22.00	1.069	23.39	0.746	−1.622 (17)	>0.05	0.37
	Group C	21.16	0.856	23.68	0.617	−4.800 (18)	<0.05 *	0.75
A	Group A	26.00	1.495	25.38	1.360	0.478 (23)	>0.05	0.10
	Group B	28.00	1.478	24.89	1.625	2.276 (17)	<0.05 *	0.48
	Group C	25.26	1.691	25.26	1.665	0.000 (18)	>0.05	0
B	Group A	16.13	1.089	16.88	1.165	−0.725 (23)	>0.05	0.15
	Group B	15.06	1.272	16.50	1.353	−1.369 (17)	>0.05	0.32
	Group C	17.89	1.546	17.21	1.337	0.492 (18)	>0.05	0.12

Group A—Intervention with Christian spirituality; Group B—Intervention without spirituality; Group C—Control group. EP—Expected prejudice; R—Religiosity; Sy—Spirituality; Ge—Gratitude; Sn—Satisfaction; Gs—Goals; A—Avoidance; B—Benevolence. * Statistically significant results are considered when *p* ≤ 0.05.

## Appendix A

**Table A1:** Details of each workshop session.

	Group A	Group B
	Topic	
	With a Christian spiritual approach	No spiritual focus
	Background music (Gregorian chants) was played in the visualization of each session [88]	Background music (instrumental Spanish guitar) was played in the visualization of each session [89]
**Sessions**		
**1**	Welcome. Group technique: “The news” Explanation of the Intervention Sign Informed Consent Fill out Participation Form Farewell	Welcome. Group technique: “The news” Explanation of the Intervention Sign Informed Consent Fill out Participation Form Farewell
**2**	Welcome. Presentation technique: “My Card” Answer instruments Farewell	Welcome. Presentation technique: “My Card” Answer instruments Farewell
**3**	Welcome Psychoeducation on Breathing/Relaxation Techniques: Deep breathing with air retention/Controlled breathing The contribution of Christian spirituality to the subject is addressed (verses) Individual execution of techniques Clarification of doubts and comments Farewell	Welcome Psychoeducation on Breathing/Relaxation Techniques: Deep breathing with air retention/Controlled breathing Individual execution of techniques Clarification of doubts and comments Farewell
	Note: The execution of the techniques was carried out in the following way: The facilitator directed the technique with one of the participants, while the others observed how it was carried out. All participants performed the technique individually, led by the facilitator	
**4**	Welcome and briefly address the task Psychoeducation on the Technique of Positive Affirmations (PowerPoint presentation was used) The contribution of Christian spirituality to the subject is addressed (verses) Individual execution of the technique: “Positive Written Affirmations” Clarification of doubts and comments Farewell	Welcome and briefly address the task Psychoeducation on the Technique of Positive Affirmations (PowerPoint presentation was used) Individual execution of the technique: “Positive Written Affirmations” Clarification of doubts and comments Farewell
**5**	Welcome and briefly address the task Psychoeducation on the Creative Visualization Technique (PowerPoint presentation was used) The contribution of Christian spirituality to the subject is addressed (verses) Individual execution of the technique: “The pink bubble” and “The creation of his sanctuary”, also called “The safe place” Clarification of doubts and comments Farewell	Welcome and briefly address the task Psychoeducation on the Creative Visualization Technique (PowerPoint presentation was used) Individual execution of the technique: “The pink bubble” and “The creation of his sanctuary”, also called “The safe place” Clarification of doubts and comments Farewell
**6**	Welcome and briefly address the task Brief Psychoeducation on Gratitude (PowerPoint presentation was used) The contribution of Christian spirituality to the subject is addressed (verses) Techniques: Deep breathing with air retention/Controlled breathing Creative Visualization and Positive Affirmations about: Thankfulness → Creative Visualization Notebook: Personal development, Educational development, Work, Money, Lifestyle, Personal objects, Relationships, Leisure, Body, etc. Share the experience Farewell	Welcome and briefly address the task Brief Psychoeducation on Gratitude (PowerPoint presentation was used) Techniques: Deep breathing with air retention/Controlled breathing Creative Visualization and Positive Affirmations about: Thankfulness → Creative Visualization Notebook: Personal development, Educational development, Work, Money, Lifestyle, Personal objects, Relationships, Leisure, Body, etc. Share the experience Farewell
	Note: When the gratitude list is made for each of the indicated aspects, the person must visualize what is being noted, that is, the positive affirmations of gratitude	
**7**	Welcome and briefly address the task A video was used with a reflection on gratitude Brief Psychoeducation on Gratitude The contribution of Christian spirituality to the theme is addressed (verses/parables). A video was used with a reflection with spiritual content on gratitude Techniques: Deep breathing with air retention/Controlled breathing Creative Visualization and Positive Affirmations on: Thanking → Creative Visualization Notebook → Visualizing giving thanks Share the experience Farewell	Welcome and briefly address the task A video was used with a reflection on gratitude Brief Psychoeducation on Gratitude Techniques: Deep breathing with air retention/Controlled breathing Creative Visualization and Positive Affirmations on: Thanking → Creative Visualization Notebook → Visualizing giving thanks Share the experience Farewell
**8**	Welcome and briefly address the task A video was used that deals with the book “Man’s Search for Meaning” by Viktor Frankl Brief Psychoeducation on Meaning of Life (PowerPoint presentation was used) The contribution of Christian spirituality to the subject is addressed (verses). A video was used that deals with the proof of the existence of God Techniques: Deep breathing with air retention/Controlled breathing Creative Visualization and Positive Affirmations about: Meaning of Life → Goal Setting Share the experience Farewell	Welcome and briefly address the task A video was used that deals with the book “Man’s Search for Meaning” by Viktor Frankl Brief Psychoeducation on Meaning of Life (PowerPoint presentation was used) Techniques: Deep breathing with air retention/Controlled breathing Creative Visualization and Positive Affirmations about: Meaning of Life → Goal Setting Share the experience Farewell
**9**	Welcome and briefly address the task Brief Psychoeducation on Meaning of Life A video was used that deals with a father who participates with his son in a triathlon competition. The son has a physical disability. The message has spiritual content The contribution of Christian spirituality to the subject is addressed (verses). Techniques: Deep breathing with air retention/Controlled breathing Creative Visualization and Positive Affirmations about: Meaning of Life → The ideal situation → The pink bubble Share the experience Farewell	Welcome and briefly address the task Brief Psychoeducation on Meaning of Life An animated short was used that deals with a boy with a degenerative disease and a girl who helps the boy at school, both manage to give meaning to their lives through a painful experience Techniques: Deep breathing with air retention/Controlled breathing Creative Visualization and Positive Affirmations about: Meaning of Life → The ideal situation → The pink bubble Share the experience Farewell
**10**	Welcome and briefly address the task Brief Psychoeducation on Forgiveness (PowerPoint presentation was used) The contribution of Christian spirituality to the subject is addressed (verses). It reflects on an interview with a popular singer who talks about his Christian faith. In addition, it reflects on a Christian song that speaks of God’s grace and forgiveness Techniques: Deep breathing with air retention/Controlled breathing Creative Visualization: “The creation of his sanctuary”, also called “The safe place” Creative Visualization and Positive Affirmations about: Forgiving Share the experience Farewell	Welcome and briefly address the task Brief Psychoeducation on Forgiveness (PowerPoint presentation was used) Techniques: Deep breathing with air retention/Controlled breathing Creative Visualization: “The creation of his sanctuary”, also called “The safe place” Creative Visualization and Positive Affirmations about: Forgiving Share the experience Farewell
**11**	Welcome and briefly address the task Brief Psychoeducation on Forgiveness The contribution of Christian spirituality to the subject is addressed (verses). It reflects on the parable about the pharisee and the publican Techniques: Deep breathing with air retention/Controlled breathing Creative Visualization: “The creation of his sanctuary”, also called “The safe place” Creative Visualization and Positive Affirmations about: Forgiving Share the experience Farewell	Welcome and briefly address the task Brief Psychoeducation on Forgiveness He reflects on a story that deals with forgiveness Techniques: Deep breathing with air retention/Controlled breathing Creative Visualization: “The creation of his sanctuary”, also called “The safe place” Creative Visualization and Positive Affirmations about: Forgiving Share the experience Farewell
**12**	Welcome and briefly address the task Answer instruments A film is projected that addresses the topics developed during the workshop Feedback on the intervention Farewell and Agape	Welcome and briefly address the task Answer instruments A film is projected that addresses the topics developed during the workshop Feedback on the intervention Farewell and Banquet

Applies to Group A and Group B of elder and youth. Older adults: Group A/Intervention with Christian spirituality *n* = 13; Group B/Intervention without spirituality *n* = 18. Young adults: Group A/Intervention with Christian spirituality *n* = 24; Group B/Intervention without spirituality *n* = 18.
